# Supplementary material for: Metabolic Profile and Metabolite Analyses in Extreme Weight Responders to Gastric Bypass Surgery
Source: Metabolites. 2022 May 6;12(5):417. doi: 10.3390/metabo12050417 (PMC9147451; doi:10.3390/metabo12050417)
Supplement: Supplementary file 1 [file metabolites-12-00417-s001.zip › metabolites-1685419-supplementary.pdf]

Supplemental Material

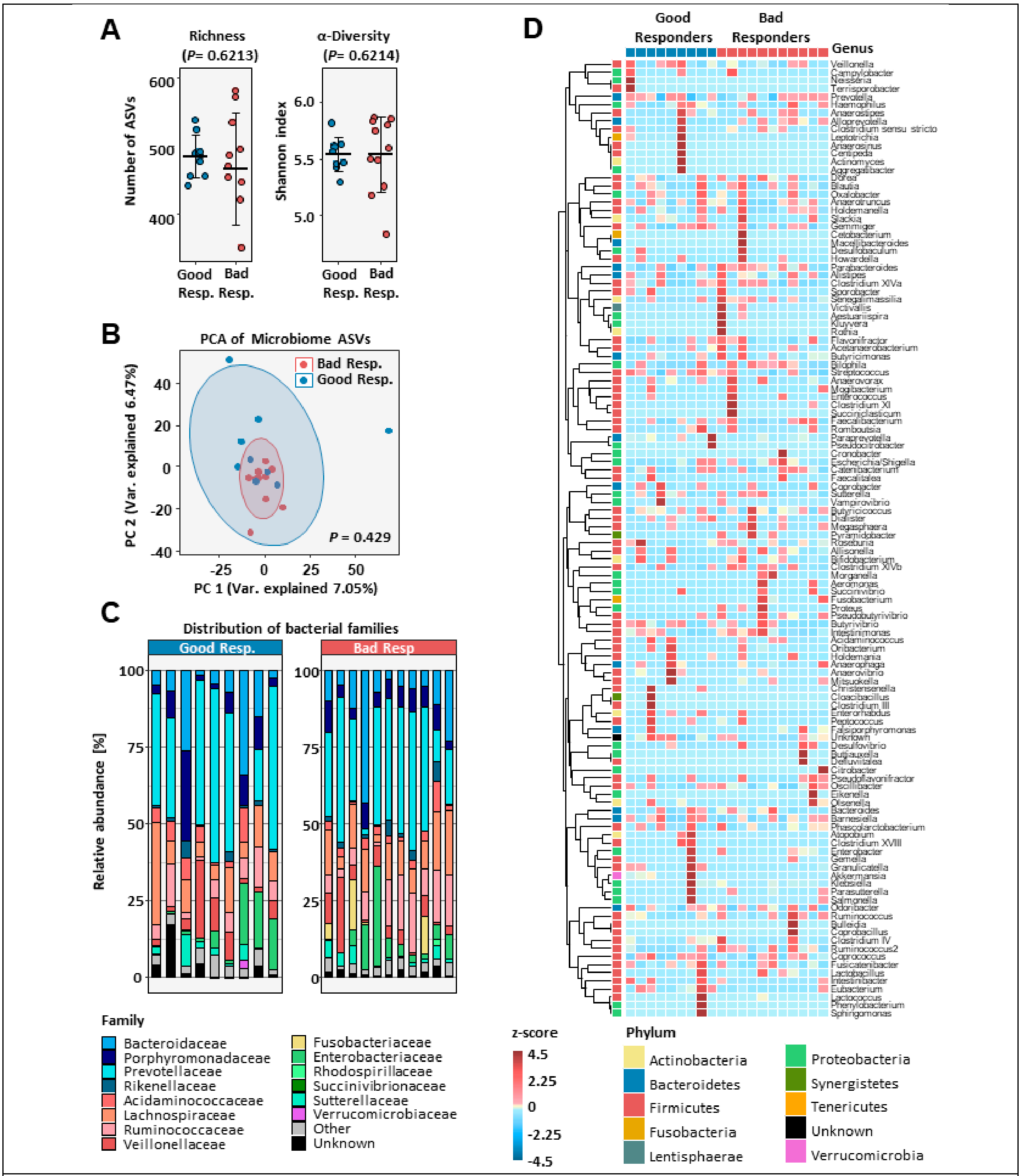

Supplemental Figure S1: Analysis of the microbiome taxonomic community structure of good responders and bad responders to RYGB surgery. In panel (A), richness (i.e. number of amplicon sequencing variants /ASV) and alpha-diversity based on the Shannon index calculated using the distribution of ASVs is depicted, with significance calculated by Kruskal-Wallis test. (B) depicts the beta-diversity or diversity between samples using principal component with PERMANOVA to calculate significance of difference between the two groups. (C) reveals the distribution of microbial families in each sample and (D) depicts the relative abundance based on z-scores of microbial genera in each sample.

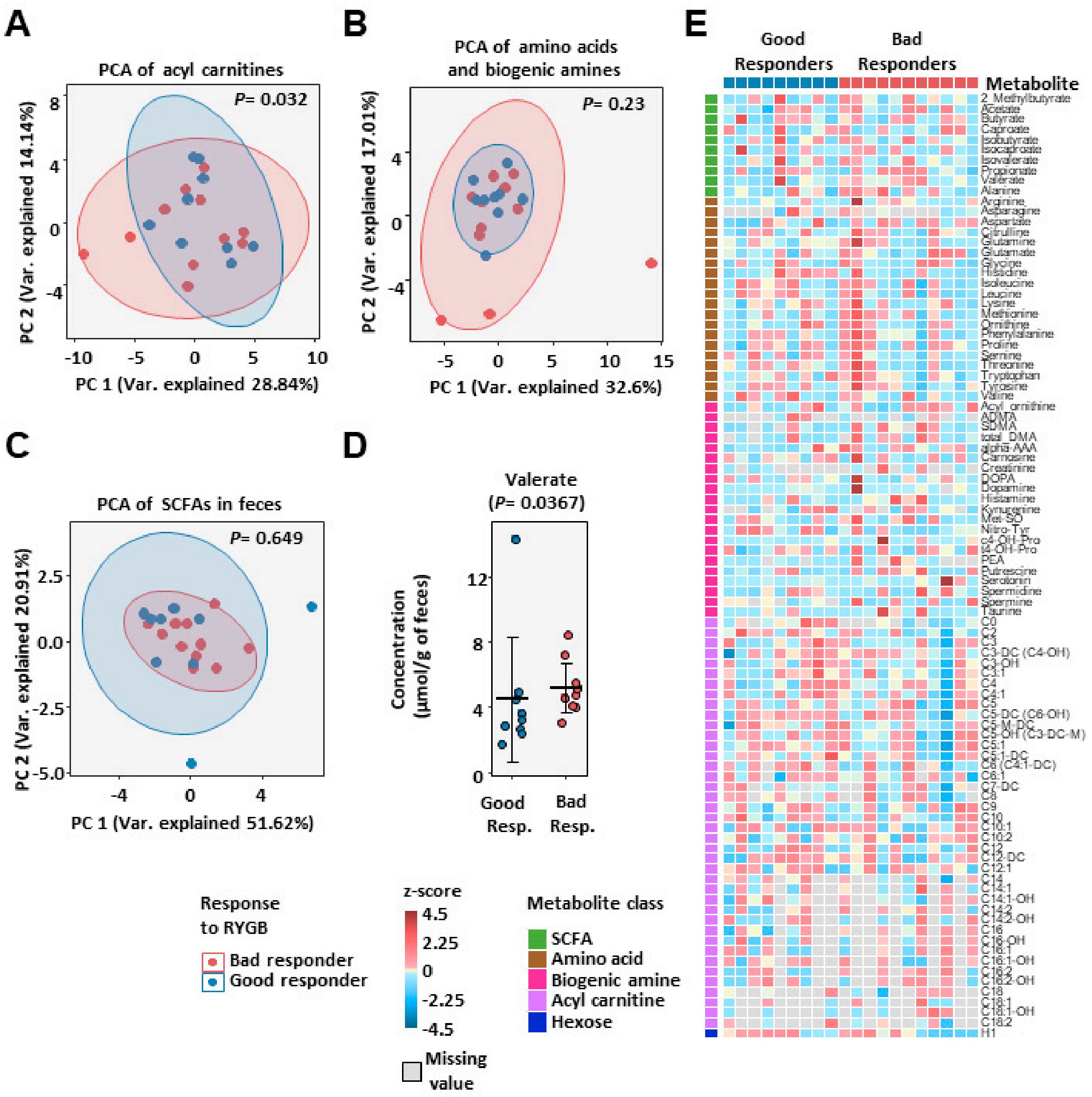

Supplemental Figure S2: Analysis of fecal metabolites. Beta-diversity analysis based on principal component analysis (PCA) of acyl carnitines (A), amino acids and biogenic amines (B), as well as short chain fatty acid (C) concentrations, with statistics calculated by PERMANOVA. (D) depicts valerate, the sole significantly different SCFA in the fecal samples between good and bad responders to RYGB, as calculated by Kruskal-Wallis test. (E) depicts relative abundance (z-scores) of metabolites measured.
